# GCompip: a pipeline for estimating the gene abundance in microbial communities

**DOI:** 10.1093/bioadv/vbaf207

**Published:** 2025-08-29

**Authors:** Xiang Zhou, Qiushuang Li, Shizhe Zhang, Wenxing Wang, Rong Wang, Xiumin Zhang, Zhiliang Tan, Min Wang

**Affiliations:** State Key Laboratory of Forage Breeding-by-Design and Utilization, National Engineering Laboratory for Pollution Control and Waste Utilization in Livestock and Poultry Production, Institute of Subtropical Agriculture, Chinese Academy of Sciences, Changsha, Hunan, 410125, China; University of Chinese Academy of Sciences, Beijing, 100049, China; State Key Laboratory of Forage Breeding-by-Design and Utilization, National Engineering Laboratory for Pollution Control and Waste Utilization in Livestock and Poultry Production, Institute of Subtropical Agriculture, Chinese Academy of Sciences, Changsha, Hunan, 410125, China; State Key Laboratory of Forage Breeding-by-Design and Utilization, National Engineering Laboratory for Pollution Control and Waste Utilization in Livestock and Poultry Production, Institute of Subtropical Agriculture, Chinese Academy of Sciences, Changsha, Hunan, 410125, China; University of Chinese Academy of Sciences, Beijing, 100049, China; State Key Laboratory of Forage Breeding-by-Design and Utilization, National Engineering Laboratory for Pollution Control and Waste Utilization in Livestock and Poultry Production, Institute of Subtropical Agriculture, Chinese Academy of Sciences, Changsha, Hunan, 410125, China; University of Chinese Academy of Sciences, Beijing, 100049, China; State Key Laboratory of Forage Breeding-by-Design and Utilization, National Engineering Laboratory for Pollution Control and Waste Utilization in Livestock and Poultry Production, Institute of Subtropical Agriculture, Chinese Academy of Sciences, Changsha, Hunan, 410125, China; State Key Laboratory of Forage Breeding-by-Design and Utilization, National Engineering Laboratory for Pollution Control and Waste Utilization in Livestock and Poultry Production, Institute of Subtropical Agriculture, Chinese Academy of Sciences, Changsha, Hunan, 410125, China; State Key Laboratory of Forage Breeding-by-Design and Utilization, National Engineering Laboratory for Pollution Control and Waste Utilization in Livestock and Poultry Production, Institute of Subtropical Agriculture, Chinese Academy of Sciences, Changsha, Hunan, 410125, China; University of Chinese Academy of Sciences, Beijing, 100049, China; State Key Laboratory of Forage Breeding-by-Design and Utilization, National Engineering Laboratory for Pollution Control and Waste Utilization in Livestock and Poultry Production, Institute of Subtropical Agriculture, Chinese Academy of Sciences, Changsha, Hunan, 410125, China; University of Chinese Academy of Sciences, Beijing, 100049, China

## Abstract

**Motivation:**

Gene abundance in metagenome datasets is commonly represented in terms of Counts or Copies Per Million. However, above term lack the consideration of the size of the microbial communities. To reflect the gene abundance in the microbial communities (GAM), GCompip, a comprehensive pipeline for estimating GAM, was developed based on specialized universal single copy genes (USCG) database, stringent alignment parameters, and rigorous filtering criteria.

**Results:**

GCompip showed high specificity without compromising computational efficiency, and improved the precision of downstream GAM estimations across diverse six ecological environments (i.e. human gut, rumen, freshwater, marine, hydrothermal sediment, and glacier). In contrast, the comparative annotation tools (i.e. KofamScan, eggNOG-mapper and HUMAnN3) showed larger error intervals, higher susceptibility to false positives, or overestimation of USCG abundance, primarily due to more relaxed thresholds, multifamily matches, or less stringent alignment settings. To facilitating the applicability of GCompip, we provided both Linux command line and R package versions. Overall, this GCompip presented an accurate, robust, user-friendly, and efficient computational pipeline designed to calculate GAM using metagenomic sequencing data. The developed pipeline makes it accessible to researchers seeking to evaluate the metabolic capabilities of microbial communities, and improve the capacity of interpreting metagenomic data related to microbial communities.

**Availability and implementation:**

GCompip package source code and documentation are freely available for download at https://github.com/XiangZhouCAS/GCompip. A separate Linux command line version is available at https://github.com/XiangZhouCAS/GCompip_onlinux.

## 1 Introduction

Microbial communities perform a wide array of essential functions in maintaining ecosystem integrity, regulating biogeochemical cycles, and supporting the health of higher organisms. The critical importance of understanding these microbial ecosystems is underscored by their central roles in ecological processes (McGill *et al.* 2007). Microbial metagenome collects the DNA of all the microorganisms in a particular environment, as the price of sequencing and arithmetic resources decreases each year, the potential of metagenomics technologies to investigate microbial diversity and function in any ecosystem has increased rapidly. It has become a key technology in exploring the distribution, interactions, and assembly processes of microbial communities (Skillings and Hooks 2019).

For analysis of metagenomic data, gene abundance is usually the focus of researchers. Counts or copies per million (CPM) are always employed to be normalized, as differential sequencing depths happen between samples (Vallejos *et al.* 2017). Reads per kilobase per million mapped reads (RPKM)/fragments per kilobase per million (FPKM) normalize gene length based on CPM, whereas transcripts per million mapped reads (TPM) further normalizes gene length and sequencing depth based on RPKM/FPKM (Zhao *et al.* 2021). As a result, the sum of TPM of all genes in each sample is 106, which can be more suitable for comparison between different samples. Although above calculation method has been widely used in metagenomic data analysis, it lacks the consideration of the size of the microbial communities and cannot fully reflect the gene abundance in the microbial communities (GAM) (Li *et al.* 2024). Such a metric would offer a more granular and context-specific understanding of how genes distributed across different microbial communities and ecological niches, thereby improving our capacity to interpret metagenomic data in relation to microbial communities. Developing robust procedures to estimate GAM could thus be a pivotal step in advancing the field of microbial ecology and metagenomics.

Universal single copy genes (USCGs) are marker genes that occur once in almost every genome (Wang *et al.* 2022). The ratio of the specific gene to the USCGs can be employed to calculate the GAM of the particular function. Due to its conservation and uniqueness, USCG has always been regarded as a potential phylogenetic marker by researchers, and can be employed to estimate the overall size of microbial communities (Darling *et al.* 2014). Currently, the software for annotating gene includes HUMAnN3 (Beghini *et al.* 2021), KOfamScan (Aramaki *et al.* 2020), and eggNOG-mapper (Cantalapiedra *et al.* 2021). HUMAnN3 calculates the reads counts of the gene family by aligning the sequence to a known protein database (e.g. Uniref 90), and uses the maximum parsimony method to assign the gene to the metabolic pathway, and finally outputs the coverage and read counts information of the pathway; KOfamScan is a gene annotation tool based on KEGG orthology (KO) and hidden Markov model (HMM); eggNOG-mapper is based on orthology groups (OGs) and uses the eggNOG database to annotate gene set functions. However, due to the wide threshold setting or complex calculation process, the outputs of those software for calculating USCG read counts can widely vary and lead to inconsistent results.

Here, we introduced a comprehensive approach, GCompip (pipeline of gene abundance calculation in microbial communities), specifically designed to estimate GAM using metagenomic sequencing data based on USCGs. The outputs of GCompip were then evaluated by comparing with KOfamScan, eggNOG-mapper, and HUMAnN3, when annotating USCG from metagenomic samples of six microbial ecosystems, i.e. rumen, human gut, freshwater, marine, glacier, and sediment. Finally, we used nitrate reductase subunit G (narG) as an example gene to calculate GAM. By comparing with other software, GCompip streamlined the calculation of GAM, and provides a more accurate and accessible means for evaluating the functional diversity and metabolic capabilities of microbial ecosystems.

## 2 Methods

### 2.1 Workflow summary

**Figure 1.**
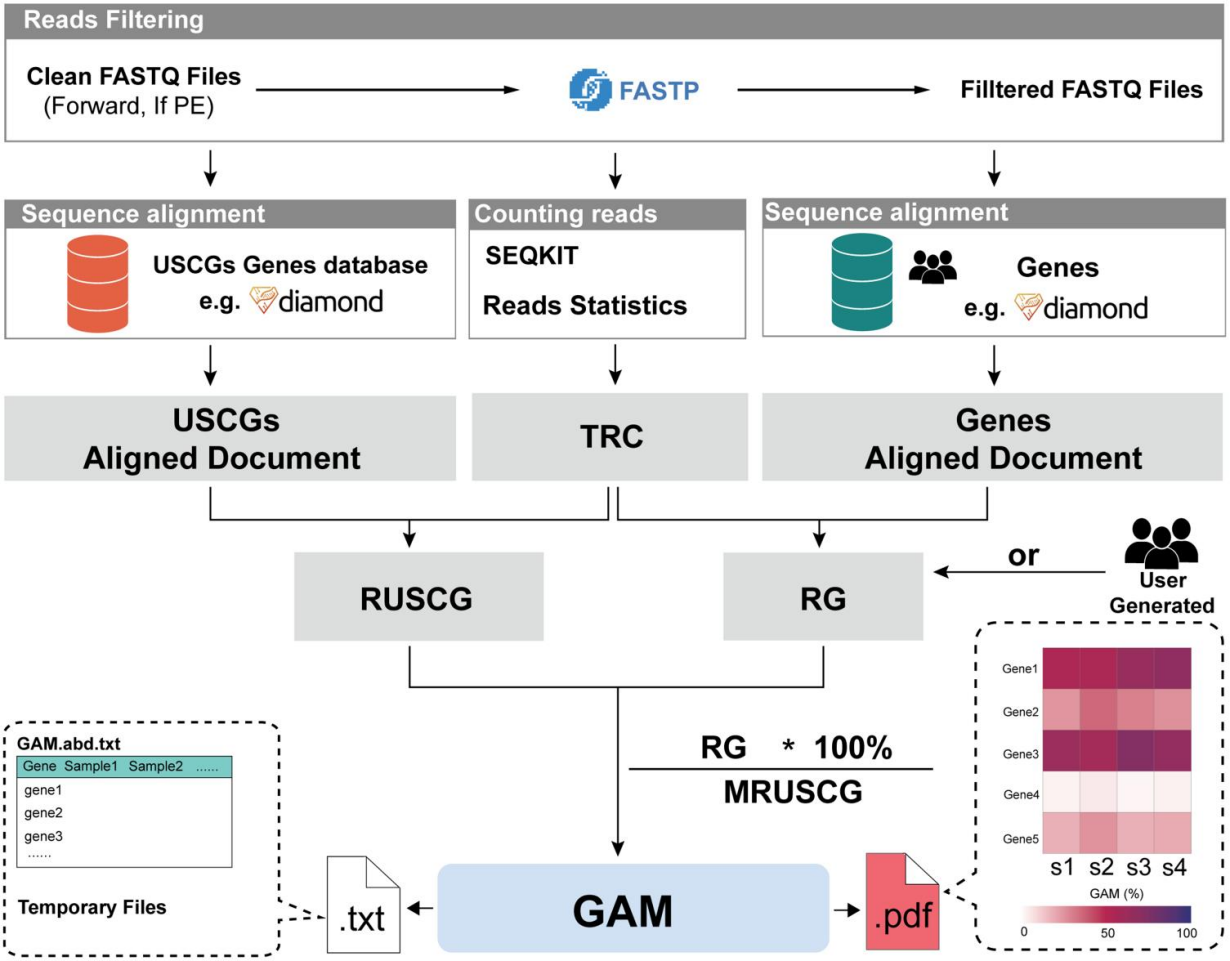
The concept of developing GCompip to calculate GAM. TRC, total read counts (in millions); USCG, universal single-copy genes; RUSCG, the read count of 14 types of USCG (in RPKM); RG, the read counts of gene (in RPKM); MRUSCG, mean of the read counts of 14 types of USCG (in RPKM); GAM, gene abundance in microbial communities (%); RPKM, reads per kilobase per million.

To estimate the GAM, the complete workflow of GCompip is shown in Fig. 1. It comprises three primary components: (i) calculating the geometric mean of the read counts of different types of USCGs (MRUSCG, in RPKM); (ii) calculating the read counts of genes (RG, in RPKM); (iii) calculating GAM (%) based on the MRUSCG and RG.

#### 2.1.1 Database building

The ribosomal protein large subunit and small subunit genes are highly conserved and have a slow evolution rate (Wu *et al.* 2013). They exist in almost all bacteria and archaea, are single copies, and thus can be used as USCG. Therefore, a total of 14 ribosomal USCGs from the Phylosift (Darling *et al.* 2014) and GTDB-Tk (Chaumeil *et al.* 2019) marker gene sets are selected to estimate the size of microbial communities. These include L6_rplF, L14b_L23e_rplN, S15P_S13e, S10_rpsJ, S12_S23, S19_rpsS, S7, L5_rplE, L11_rplK, L16_L10E_rplP, L3_rplC, S5, S2_rpsB, and L2_rplB (Table 1). Moreover, these marker genes may be present in “multiple copies” due to false positive. We then employed the geometric mean of these USCGs as the final size of the microbial communities to minimize this problem (Tsuji *et al.* 2018).

**Table 1.** The selected 14 ribosomal universal single-copy genes (USCGs) for estimate the size of microbial communities.

Gene name	KEGG orthology	COG number	In bacteria (%)	In archaea (%)
S2_rpsB	K02967	COG0052	99.50	100
S10_rpsJ	K02946	COG0051	98.51	100
L6_rplF	K02933	COG0097	99.50	100
S12_S23	K02950	COG0048	99.17	100
	K02973			
S15P_S13e	K02956	COG0184	99.84	100
	K02953			
S19_rpsS	K02965	COG0185	99.17	100
L5_rplE	K02931	COG0094	99.83	100
L11_rplK	K02867	COG0080	99.83	100
L14b_L23e_rplN	K02874	COG0093	99.34	100
	K02894			
L16_L10E_rplP	K02878	COG0197	99.67	100
	K02866			
S7	K01316	COG0049	99.67	100
L3_rplC	K02906	COG0087	99.50	100
S5	K02988	COG0098	99.50	100
L2_rplB	K02886	COG0090	99.50	100

In order to ensure the accuracy and efficiency of sequence alignment, reference sequences dereplication of 14 USCGs at 60% identity by CD-HIT (Li and Godzik 2006) (parameter: cd-hit -n 3 -M 0 -c 0.6). Because of its high precision and fast speed in short sequence alignment, DIAMOND (Buchfink *et al.* 2021) is selected as the comparison software, and the database is built first (diamond makedb, then, diamond makeidx). We have putted the USCG database at github home page of GCompip (https://github.com/XiangZhouCAS/GCompip/tree/main/database).

#### 2.1.2 Pipeline establishment

In this pipeline, we calculated GAM using only forward sequences for paired-end data, because its direction is consistent with mRNA, closer to the actual transcription product and suitable for gene sequence analysis (Yang *et al.* 2018). Firstly, quality control was performed using fastp (Chen 2023), filtering out reads shorter than 50 bp, which were considered too short to retain. We did not annotate all the genes in the sample, resulting in the inability to calculate TPM, while CPM did not consider the effect of gene length on read counts, so we selected RPKM as the read counts unit of gene community size. The total number of read counts (TRC, in millions) per sample was obtained through Seqkit (Shen *et al.* 2024), whereas both mapped read counts (MRC) and gene length of USCGs per sample were obtained by aligning the diamond database of the 14 USCGs (Buchfink *et al.* 2021). The resulting m6 file contained the length, name, and read counts of each USCG. Mapped reads were filtered to retain only those with a bitscore ≥40 and query coverage ≥80%. Then, the read counts of each USCG (RUSCG, in RPKM) were calculated by dividing the MRC of USCG by TRC and then dividing by the gene length (in bp) using Equation (1). Finally, the geometric mean of the read counts of 14 USCGs (MRUSCG, in RPKM) was calculated by Equation (2).


(1) $$\mathrm{RPKM}=\;\frac{\mathrm{MRC}\times 10^{9}}{\mathrm{TRC}\;\times \mathrm{gene}\;\mathrm{length}}$$

(2) $$\mathrm{MRUSCG}=\sqrt[{14}]{{\mathrm{RUSCG}}_{1}\times {\mathrm{RUSCG}}_{2}\times {\mathrm{RUSCG}}_{3}\times \cdots \times {\mathrm{RUSCG}}_{14}}$$

where RUSCG*i* was the read counts of USCGs; *i* (in RPKM) was L6_rplF, L14b_L23e_rplN, S15P_S13e, S10_rpsJ, S12_S23, S19_rpsS, S7, L5_rplE, L11_rplK, L16_L10E_rplP, L3_rplC, S5, S2_rpsB or L2_rplB.

Read counts of genes (RG, in RPKM) are obtained through two distinct scenarios. In the first scenario, MRC and genes’ length of genes in each sample were determined by aligning the sequencing data against a user-provided gene database. The sequence alignment produced an m6 file, which contained key information such as the gene name, sequence identity, coverage, and gene length. This alignment data was saved in .txt format for further analysis. Mapped reads were filtered based on alignment quality, with a default identity ≥50% and coverage ≥80%, whose thresholds can also be customized by the user. Then, the read counts of each gene were calculated by dividing the MRC of each gene by TRC and then dividing by the gene length using Equation (1). In this second scenario, users could supply precalculated read counts of RG values directly. To facilitate the calculation of these values from raw sequencing data, we provided a general workflow in Fig. 1, available as supplementary data at *Bioinformatics Advances* online, which guided users through the necessary steps to obtain RGs from their raw sequence data. GAM (%) is then calculated by dividing RG by MRUSCG using Equation (3) as follows:


(3) $$\mathrm{GAM}=\frac{\mathrm{RG}}{\sqrt[{14}]{{\mathrm{RUSCG}}_{1}\times {\mathrm{RUSCG}}_{2}\times {\mathrm{RUSCG}}_{3}\times \cdots \times {\mathrm{RUSCG}}_{14}}}\times 100\%$$

where RUSCGi was the read counts of USCGs; i (in RPKM) was L6_rplF, L14b_L23e_rplN, S15P_S13e, S10_rpsJ, S12_S23, S19_rpsS, S7, L5_rplE, L11_rplK, L16_L10E_rplP, L3_rplC, S5, S2_rpsB or L2_rplB.

### 2.2 Software implementation

**Figure 2.**
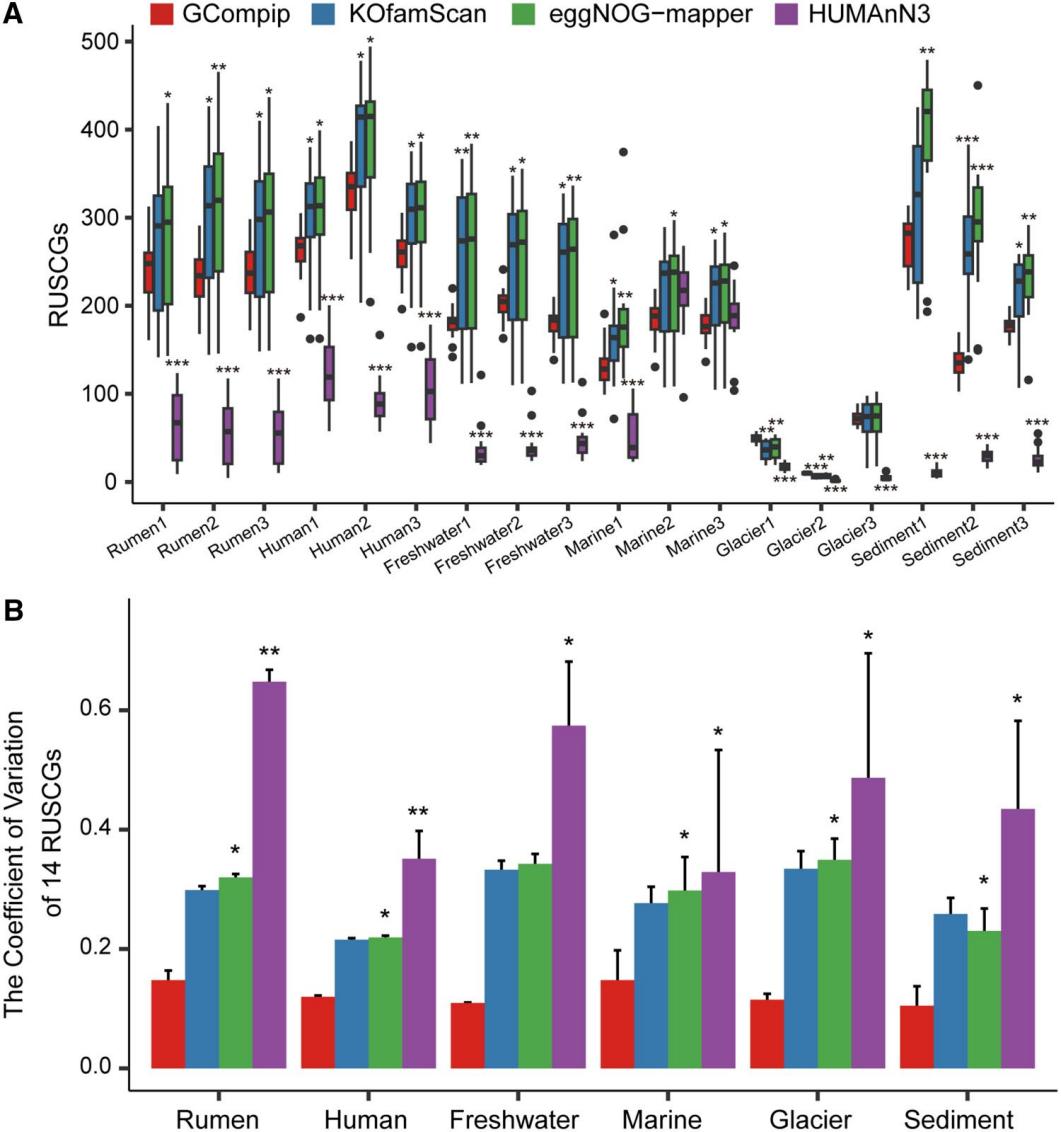
RUSCG (A) and its coefficient of variation (B) in six ecological environments calculated by four methods (i.e. GCompip, KOfamScan, eggNOG-mapper, and HUMAnN3). RUSCGs, Read counts (in RPKM) of universal single copy genes. *P < .05; **P < .01; ***P < .001 (Wilcoxon rank-sum test, same as below).

**Figure 3.**
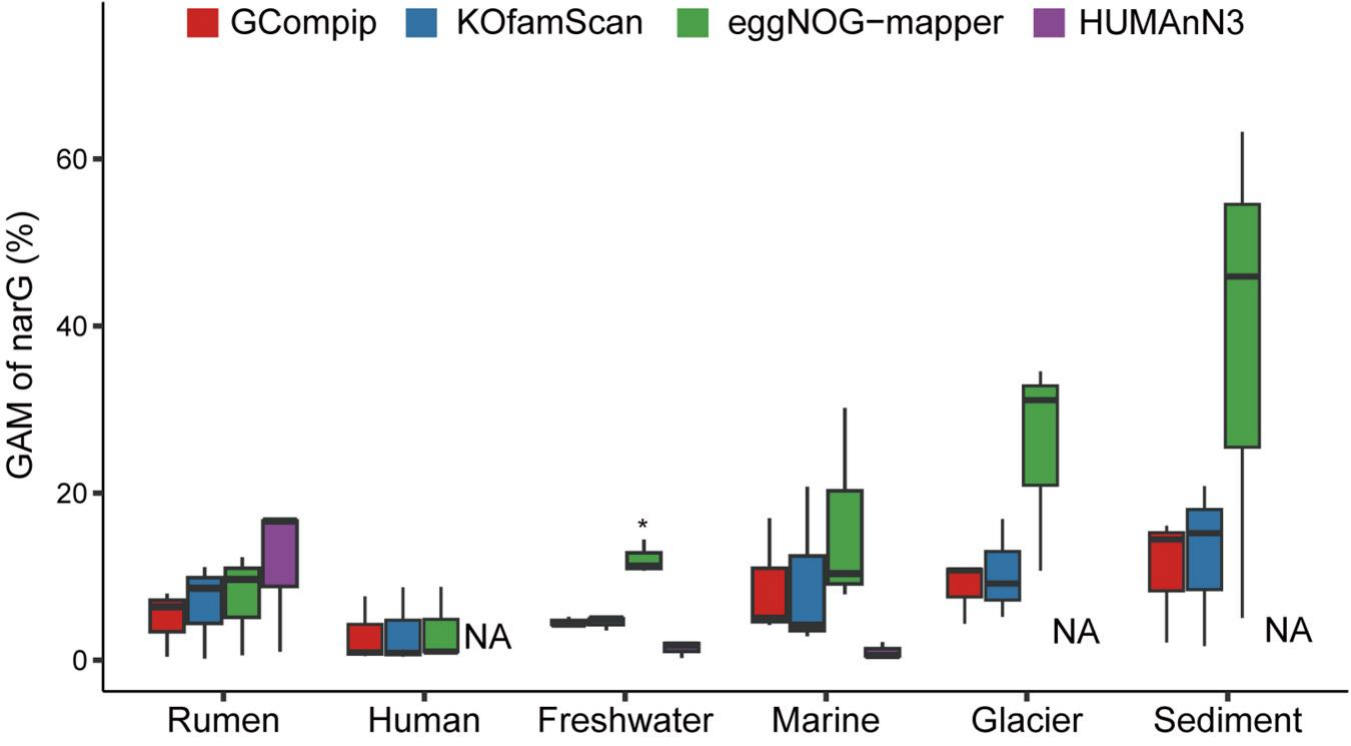
GAM of narG in six ecological environments calculated by four methods (i.e. GCompip, KOfamScan, eggNOG-mapper, and HUMAnN3). narG, nitrate reductase subunit G; GAM, gene abundance in microbial communities (%); NA, not available data. *P < .05.

To optimize usability, GCompip extracted all programming complexities and provided well-defined default parameters for key steps, such as diamond alignment. These defaults included parameters for alignment length, identity and coverage, ensuring optimal performance for most users (Table 2). However, users have the flexibility to adjust these parameters to tailor the analysis to specific needs. Regarding input requirements, users are only required to supply clean sequences with host DNA removed, along with either a gene database or pre-generated GAM tables. The pipeline can process these inputs to produce several outputs: a table of GAM values in .txt format, as well as graphical visualizations (e.g. heatmaps) in .pdf format, providing both quantitative and visual representations for GAM. In addition to these outputs, temporary files are generated during the analysis and include the seqkit statistics table for read counts, diamond alignment files for both USCGs and genes, all of which are saved by default in .txt format. These intermediate files can be retained or discarded by the user depending on their specific requirements. This design ensures a streamlined workflow while offering flexibility for customization and further exploration of the data. GCompip’s help page, more details of functions and file formats can be found at Note 1, available as supplementary data at *Bioinformatics Advances* online (Fig. 2, Table 1–3 and Figs 3–5, available as supplementary data at *Bioinformatics Advances* online, respectively).

**Table 2.** The defaults values of alignment length, identity and coverage set in GCompip.

Parameter	Description	Default
min_length	Set the minimum length required for filtering reads, the default is 50 bp.	50
filter_condition	Minimum threshold of identity and coverage in alignment.	Identity = 50
		coverage = 80

In addition to the R package version, we also provided a Linux command line version, which was consistent with the R package version to enhance the applicability of GCompip.

### 2.3 Comparison of calculations method

We selected 18 metagenomic samples collected from six ecological environments such as human gut, rumen, freshwater, marine, hydrothermal sediment, and glacier, and included multiple sequencing depths of 5–60 G, sample’s information as shown in Note 2, available as supplementary data at *Bioinformatics Advances* online (Table 4, available as supplementary data at *Bioinformatics Advances* online). GCompip, KOfamScan, eggNOG-mapper, and HUMAnN3 were employed to separately calculate the RUSCGs. The RUSCGs calculated by GCompip were lower than other three methods in samples collected from six ecological environments (Fig. 2A). We then calculated the coefficients of variation of 14 USCGs (Note 2, available as supplementary data at *Bioinformatics Advances* online), which indicated that error of RUSCG calculated by GCompip was significantly smaller than eggNOG-mapper and HUMAnN3 (Fig. 2B). NarG, a gene catalyzing the reduction of nitrate to nitrite and widely distributed in six ecological environments (Kandeler *et al.* 2006, Auclair *et al.* 2010, Rothery *et al.* 2010, Poulsen *et al.* 2014, Rasigraf *et al.* 2017, Li *et al.* 2025). Results indicated that GAM of narG calculated by GCompip can be consistently obtained in samples collected from six ecological environments, and showed lowest variations among four calculation methods (Fig. 3).

## 3 Discussion

GCompip has a specialized database of 14 USCGs. Due to the alignment parameters set as “--max-target-seqs 1 --max-hsps 1,” only the unique best alignment result is returned for each alignment. Moreover, sequences with coverage <80% and bit scores lower than 40 were removed, thereby strictly controlling the quality and quantity of alignments. Additionally, GCompip has strong representativeness of the reference sequences of USCGs based on ribosomal protein, and some new USCGs (i.e. recA) may be further incorporated to update the database of USCGs, when necessary. It can directly conduct efficient alignments with high-throughput sequences, which significantly improved the efficiency of calculating the RUSCGs.

GCompip versus KOfamScan: compared with GCompip, the RUSCG calculated by KOfamScan was higher with larger error interval. In the subsequent calculating GAM of narG, KOfamScan had a larger error than GCompip in samples collected from six ecological environments. KOfamScan has a specialized annotation database for KOs (KEGG Orthologs), uses high-quality HMM models and adaptive alignment score thresholds for comprehensive KEGG Orthologs. Although the thresholds are specifically set for each KO with effectively reduced the likelihood of annotation errors, USCGs in KOfamScan can be matched multiple KO families by the pHMM models, which can increase the probability of false positives. Additionally, KOfamScan sets thresholds by maximizing the F-value, which may sacrifice specificity to balance sensitivity, leading to incorrect KO assignments caused by the relaxed thresholds.

GCompip versus eggNOG-mapper: compared with GCompip, the RUSCG calculated by eggNOG-mapper was higher with larger annotation error interval. In the subsequent calculating GAM of narG, eggNOG-mapper had a larger annotation error than GCompip in samples collected from six ecological environments. During the DIAMOND alignment, the sensitive mode (—sensitive) and selection of the top three alignment scores (—top 3) in default setting of eggNOG-mapper can increase the probability of false positives in alignments, thereby reducing the alignment accuracy of USCGs.

GCompip versus HUMAnN3: compared with GCompip, the RUSCG calculated by HUMAnN3 was lower with a larger annotation error interval. In the subsequent calculating GAM of narG, narG in samples from marine, glacier and sediment could not be annotated by HUMAnN3. This is not the case, as narG has been widely distributed in those microbial ecosystems. Additionally, HUMAnN3 uses bowtie2 (Langmead and Salzberg 2012) and DIAMOND successively for sequence annotation. During the DIAMOND alignment, the default e-value in HUMAnN3 is set to 1.0, which is far higher than the default e-value of 0.001 in DIAMOND, resulting in an increased number of aligned sequences. Due to HUMAnN3’s read counts distribution strategy, some read counts is allocated to other genes, leading to the underestimation of the RUSCGs.

## 4 Conclusion

We present GCompip, a pipeline for calculating GAM. By comparison, we demonstrated its applicability in different microbial ecosystems. GCompip outperforms KOfamScan, eggNOG-mapper, and HUMAnN3 in RUSCG estimation by combining a specialized USCG database with stringent alignment and filtering criteria, reducing false positives while preserving efficiency. This ensures more accurate and consistent functional gene abundance estimates across diverse environments. The use of GCompip can enhance our understanding of the distribution of genes in microbial communities and improve our ability to interpret metagenomic data related to microbial communities. In addition, the Linux command line version we provide greatly expands its applicability. In the future, we plan to further update the USCG database and increase the types of alignment software (e.g. hmmsearch, blast, etc.) to further increase the accuracy and applicability of GCompip.

## Supplementary Material

vbaf207_Supplementary_Data

## Data Availability

The data underlying this article are available in the article and in its online supplementary file.
