# Supplementary material for: GCompip: a pipeline for estimating the gene abundance in microbial communities
Source: Bioinform Adv. 2025 Aug 29;5(1):vbaf207. doi: 10.1093/bioadv/vbaf207 (PMC12460045; doi:10.1093/bioadv/vbaf207)
Supplement: vbaf207_Supplementary_Data [file vbaf207_supplementary_data.docx]

**Supplementary information for**

**GCompip: A pipeline for estimating the gene abundance in microbial communities**

Xiang Zhou^1,2^,Qiushuang Li^1^,Shizhe Zhang^1,2^,Wenxing Wang^1,2^,Rong Wang^1^, Xiumin Zhang^1^, Zhiliang Tan^1,3^, Min Wang^1,2,^*

^1^State Key Laboratory of Forage Breeding-by-Design and Utilization, National Engineering Laboratory for Pollution Control and Waste Utilization in Livestock and Poultry Production, and Hunan Provincial Key Laboratory of Animal Nutritional Physiology and Metabolic Process, Institute of Subtropical Agriculture, Chinese Academy of Sciences, Changsha, Hunan, China.

^2^University of Chinese Academy of Sciences, Beijing, China.

*Corresponding author: Min Wang, Email: [mwang@isa.ac.cn](mailto:mwang@isa.ac.cn)

**This file includes:**

Supplementary Figure S1 to S5

Supplementary Table S1 to S5

Supplementary Note 1

Supplementary Note 2

**Supplementary Information Text:**

**Supplementary Figures:**

**Figure S1.** Read counts (in RPKM) of gene set’s workflow.

**Figure S2.** Help page of GCompip.

**Figure S3.** Format of filter condition.

**Figure S4.** Format of geneset.RPKM.txt.

**Figure S5.** Format of RUSCG.txt.

**Supplementary Table:**

**Table S1.** Parameters setting of diamond_GAM.

**Table S2.** Parameters setting of uscg_rpkm.

**Table S3.** Parameter setting of uscg_rpkm2GAM.

**Table S4.** Information of samples.

**Table S5.** The coefficient of variation of 14 USCGs’ RPKM for each sample calculated by GCompip, KOfamScan, eggNOG-mapper and HUMAnN3.

**
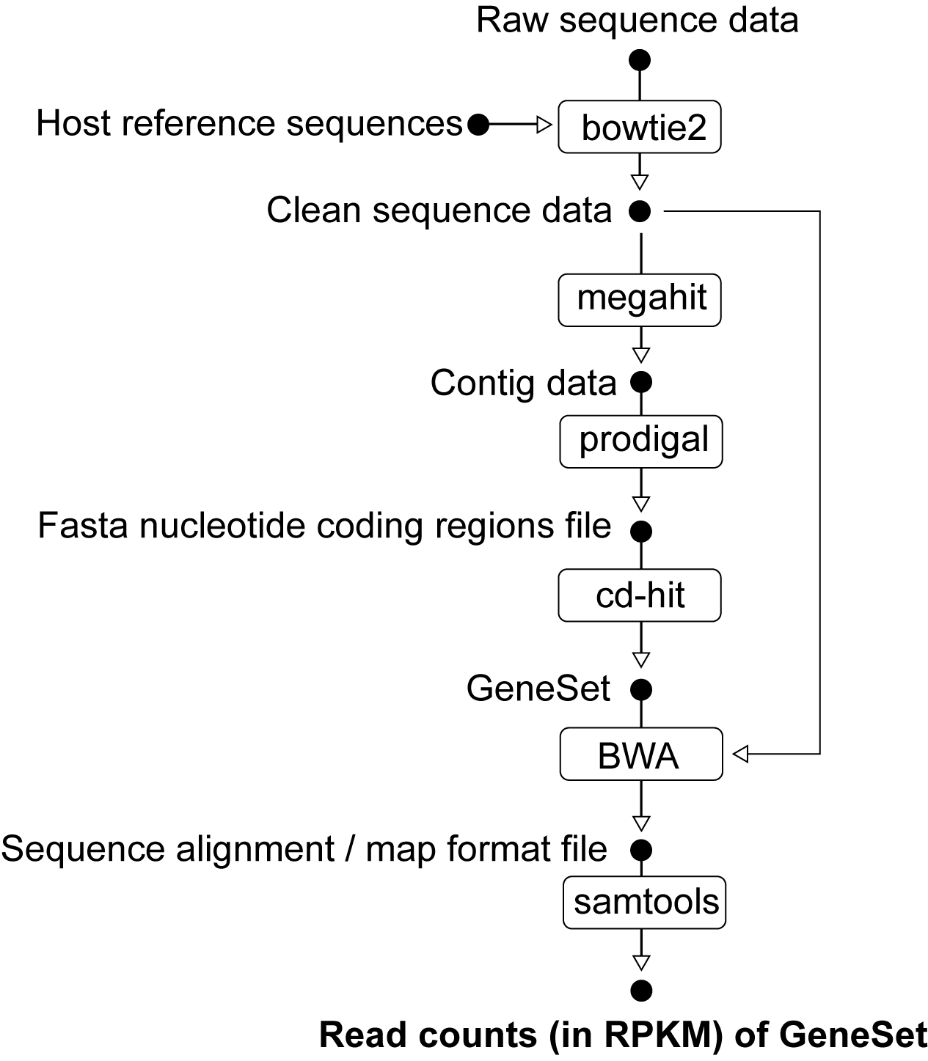
Supplementary Figure S1.** Read counts (in RPKM) of gene set’s workflow. These softwares are required: bowtie2 (Langmead and Salzberg, 2012), megahit (Li, et al., 2015), prodigal (Hyatt, et al., 2010), cd-hit (Li and Godzik, 2006), bwa (Li and Durbin, 2009), seqkit (Shen, et al., 2016) and samtools (Danecek, et al., 2021).

**Step1: Remove host’s sequence.**

bowtie2 -p #threads -x hostgenome -1 sample_name.raw.1.fq.gz -2 sample_name.raw.2.fq.gz -S sample_name.sam --un-conc sample_name.raw.clean.fq

**Step2: Assemble contigs from sequences. (rename_fasta.sh at the end of the document)**

megahit -1 sample_name.clean.1.fq.gz -2 sample_name.clean.2.fq.gz --min-contig-len #length -t #threads -o sample_name

sh rename_fasta.sh final.contigs.fa sample_name sample_name.contig.ok.fa

**Step3: Predicting genes from contigs.**

prodigal -i sample_name.contig.ok.fa -f gff -o sample_name.gff -p meta -q -d sample_name.temp.orf.ffn -a sample_name.temp.orf.faa

perl gene-filter.pl sample_name.temp.orf.ffn sample_name.temp.orf.faa sample_name.orf.ffn sample_name.orf.faa

**Step4: Generate non-redundant representative sequences.**

cd-hit-est -i sample_name.orf.ffn -o sample_name.geneSet.ffn -n 9 -c 0.95 -G 0 -M 0 -d 0 -aS 0.9 -r 0 -T 40

Cat *geneSet.ffn >> DNA.geneSet.tmp.ffn

cd-hit-est -i DNA.geneSet.tmp.ffn -o DNA.geneSet.ffn -n 9 -c 0.95 -G 0 -M 0 -d 0 -aS 0.9 -r 0 -T 40

**Step5: Clean sequences aligning with gene set.**

bwa index DNA.geneSet.ffn

bwa mem -t #threads -M -R '@RG\tID:$i\tSM:$i\tLB:$i\tPL:Illumina\tPI:$i' DNA.geneSet.ffn sample_name.clean.1.fq.gz sample_name.clean.2.fq.gz > sample_name.sam

**Step6: Calculate genes’ RPKM in gene set. (rpkm.r at the end of the document)**

# Counting total read counts (TRC) and length of genes.

perl read_counts.pl DNA.geneSet.ffn sample_name.sam sample_name.sam.abd

sh ./list.sh

perl abd.merge.pl list TPM.txt reads_counts.txt

# Calculate genes’ RPKM in gene set.

cut -f 2 sample.sam.abd (only need one sample to generate it) > genelength.txt

sed -i '1d' genelength.txt

Rscript rpkm.r -i reads_counts.txt -g genelenth.txt -o RPKM.txt

**rename_fasta.sh:**

#!/bin/bash

if [ "$#" -ne 3 ]; then

echo "Usage: $0 <input_fasta_file> <prefix> <output_file>"

exit 1

fi

input_file="$1"

prefix="$2"

output_file="$3"

awk -v prefix="$prefix" '

BEGIN { counter = 1 }

/^>/ {print ">" prefix "_" counter;counter++}

!/^>/ {print}

' "$input_file" > "$output_file"

echo "Renamed sequences have been written to $output_file"

**gene-filter.pl**

echo "Renamed sequences have been written to $output_file"

die "perl input.ffn input.faa output.ffn output.faa\n" if(@ARGV!=4);

open IA, "$ARGV[0]" or die "can not open file: $ARGV[0]\n";

open IB, "$ARGV[1]" or die "can not open file: $ARGV[1]\n";

open OA, ">$ARGV[2]" or die "can not open file: $ARGV[2]\n";

open OB, ">$ARGV[3]" or die "can not open file: $ARGV[3]\n";

$/=">";<IA>;<IB>;

while($line=<IA>){

chomp $line;

my @ele=split /\n/,$line;

my $cds="";

my @inf=split /\s+/,$ele[0];

for(my $i=1;$i<=$#ele;$i++){

$cds.=$ele[$i];

}

$line=<IB>;

chomp $line;

@ele=split /\n/,$line;

my $aa="";

for(my $i=1;$i<=$#ele;$i++){

$aa.=$ele[$i];

}

if(length($cds)>100){

$aa=~s/\*//g;

print OA ">$inf[0]\n$cds\n";

print OB ">$inf[0]\n$aa\n";

}

}

close IA;

close OA;

close IB;

close OB;

**read_counts.pl**

use FindBin qw($Bin);

die "perl uniqGene-ProfilebySoap.pl gene.fa sam out\n" if(@ARGV!=3);

my ($line,@inf,%genelen,%abu,$sum);

open IN, "$ARGV[0]" or die "can not open $ARGV[0]\n";

$/=">";

<IN>;

while($line=<IN>){

chomp $line;

@inf=split /\n/,$line;

my @id=split /\s/,$inf[0];

my $seq="";

for(my $i=1;$i<=$#inf;$i++){

$seq.=$inf[$i];}

$genelen{$id[0]}=length($seq);

$abu{$id[0]}=0;}

close IN;

$/="\n";

#sam results

if($ARGV[1]=~/\.bam$/){

open IN,"$Bin/samtools view $ARGV[1]|" or die "can not open $ARGV[1]\n";

}

else{

open IN,"$ARGV[1]" or die "can not open $ARGV[1]\n";

}

while($line=<IN>){

chomp $line;

@inf=split /\t/,$line;

next if($line=~/^@/ || $inf[2] eq "*" ||$inf[5] eq "*");

my ($m,$l)=(0,0);

my @ele=split /[A-Z]/,$inf[5];

for(my $i=0;$i<=$#ele;$i++){

$l+=$ele[$i];

}

my $mn=$inf[5]=~s/M/M/g;

if($mn==1){

$inf[5]=~/(\d+)M/;

$m=$1;

}

if($mn==2){

$inf[5]=~/(\d+)M\d+\w(\d+)M/;

$m+=$1+$2;

}

if($mn==3){

$inf[5]=~/(\d+)M\d+\w(\d+)M\d+\w(\d+)M/;

$m+=$1+$2+$3;

}

# print "$inf[5]\t$m\t$l\n";

if($m>=50 && ($m/$l)>0.95){

$sum++;

$abu{$inf[2]}++;

}

}

close IN;

open OA, ">$ARGV[2]" or die "can not open $ARGV[2]\n";

print OA "GeneID\tGeneLen\tReadsNum\tAbundance\tRelativeAbundance\tTotalAbundance\n";

my $temp=0;

foreach my $i (sort keys %genelen){

$temp+=$abu{$i}/$genelen{$i}/$sum;

}

foreach my $i (sort keys %genelen){

printf OA "$i\t%d\t%ld\t%.8e\t%.8e\t%.8e\n",$genelen{$i},$abu{$i},$abu{$i}/$genelen{$i}/$sum,$abu{$i}/$genelen{$i}/$sum/$temp,$temp;

}

close OA;

**list.sh**

close OA;

for i in sample_name1 sample_name2 sample_name3 ...

do

echo "$i $i.sam.abd" >> list

done

**abd.merge.pl**

die "perl Profile-Merge.pl sam.list out.profile out.read \n" if(@ARGV!=3);

my ($line,@inf,%ff,@sample);

open IN, "$ARGV[0]" or die "can not open $ARGV[0]\n";

open OA, ">$ARGV[1]" or die "can not open $ARGV[1]\n";

open OB, ">$ARGV[2]" or die "can not open $ARGV[2]\n";

print OA "GeneID"; print OB "GeneID";

while($line=<IN>){

chomp $line; @inf=split /\s+/,$line;

print OA "\t$inf[0]"; print OB "\t$inf[0]";

push @sample,$inf[0];

open $ff{$inf[0]}, "$inf[1]" or die "can not open $inf[1]\n";

my $temp=$ff{$inf[0]};<$temp>;}

print OA "\n"; print OB "\n"; close IN;

open IN, "$inf[1]" or die "can not open $inf[1]\n";

<IN>;

while($line=<IN>){

chomp $line;@inf=split /\t/,$line;

print OA "$inf[0]"; print OB "$inf[0]";

for(my $j=0;$j<=$#sample;$j++){

my $temp=$ff{$sample[$j]};

$line=<$temp>;chomp $line;@inf=split /\t/,$line;

printf OA "\t%.6f",$inf[4]*1000000;

print OB "\t$inf[2]";}

print OA "\n"; print OB "\n";}

close OA; close IN; close OB;

**rpkm.r:**

if(!require(optparse,quietly = TRUE)){

install.packages("optparse")

library(optparse)

}else{

library(optparse)}

if(!require(data.table,quietly = TRUE)){

install.packages("data.table")

library(data.table)

}else{

library(data.table)}

option_list <- list(

make_option(c("--reads_count","-i"),type = "character",

default = F,

help = "Please set the directory of reads count file."),

make_option(c("--gene_length","-l"),type = "character",

default = F,

help = "Please set the directory of gene_length file"),

make_option(c("--output","-o"),type = "character",

default = F,

help = "Please set the result name's prefix."))

opt_parser <- OptionParser(

usage = "usage: comts rpkm [option]",

option_list = option_list,

add_help_option = TRUE,

prog = NULL,

description = "To calculate the RPKM abundance of geneset.")

opt <- parse_args(opt_parser)

reads_count <- opt$reads_count

gene_length <- opt$gene_length

output <- opt$output

exprSet <- read.table(reads_count,header=T,row.names=1)

lengths <- fread(gene_length)

lengths <- as.vector(unlist(lengths))

total_count<- colSums(exprSet)

rpkm <- t(do.call(rbind,

lapply(1:length(total_count),

function(i){

10^9*exprSet[,i]/lengths/total_count[i]

}) ))

rownames(rpkm) <- rownames(exprSet)

colnames(rpkm) <- colnames(exprSet)

rpkm<-as.data.frame(rpkm)

write.table(rpkm,output,quote=F,sep = "\t")

**Supplementary Note 1**

We use R language to realize the process of GAM computing by integrating the above software and packages such as magrittr (https://magrittr.tidyverse.org, version number : 2.0.3), dplyr (https://dplyr.tidyverse.org, version number : 1.1.4), tidyr (https://tidyr.tidyverse.org, version number : 1.3.1), data.table (https://github.com/Rdatatable/data.table, version number : 1.16.2) and ggplot2 (https://ggplot2.tidyverse.org, version number : 3.5.1). Five computing functions, diamond_GAM(), ter.diamond_GAM(), hyd.diamond_GAM(), uscg_rpkm() and uscg_rpkm2GAM(), and the Database_download() function. (More details can be seen at https://github.com/XiangZhouCAS/GCompip.git)


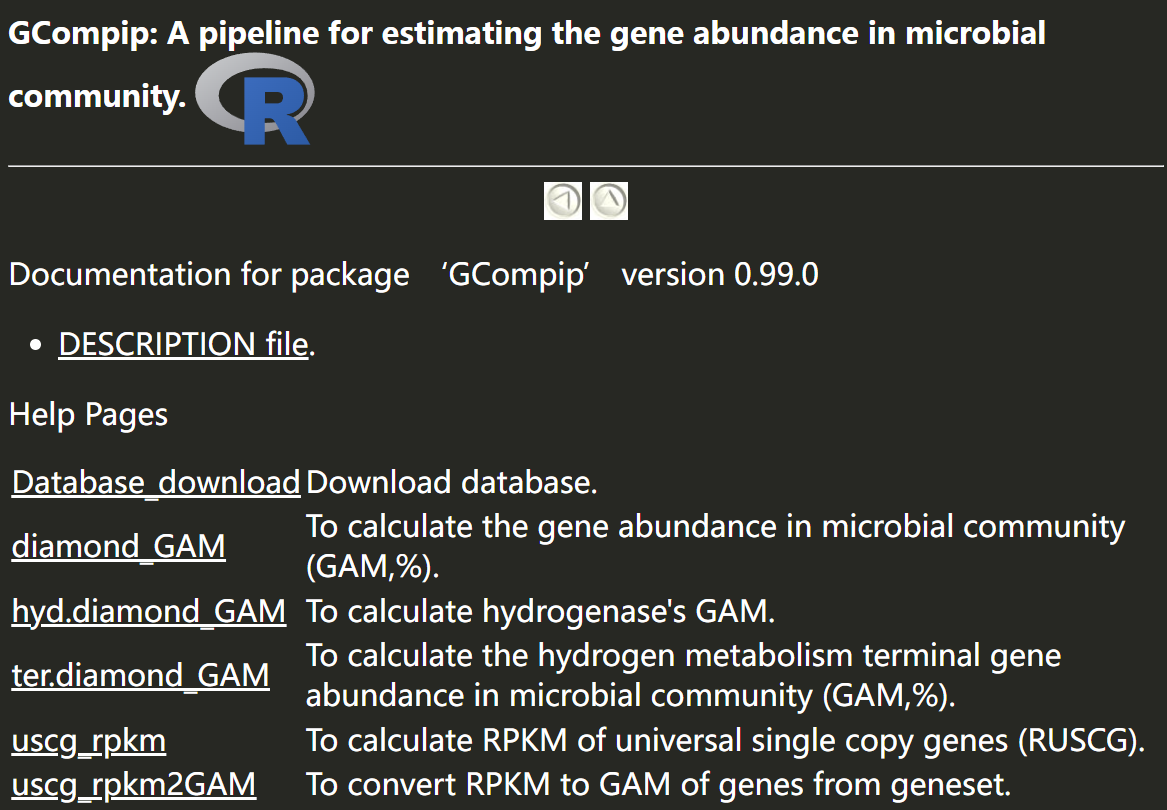
**Supplementary Figure S2.** Help page of GCompip.

The diamond_GAM() function is a general GAM calculation function. The target gene database and filtering conditions ( as shown in Supplementary Figure S3) are provided by the user. The detailed parameters of the diamond_GAM() function are shown in Supplementary Table S1.

Code example:

diamond_GAM(input_reads = "your/reads/data/sample_1.fastq", result = "sample_1", threads = 40, diamond_db = "your/database/target_genes.dmnd", USCG_db = "your/database/Ribo_14.dmnd", min_length = 75, filter_condition = "default")

**
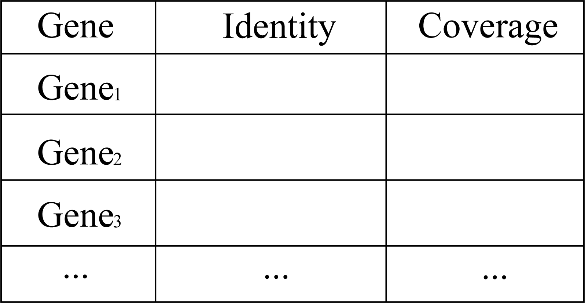
Supplementary Table S1.** Parameters setting of diamond_GAM

| **Parameter** | **Description** | **Default** |
| --- | --- | --- |
| input_reads | Set the reads to include only the forward reads if the data is paired-end (PE). | / |
| result | Set the name for the result file. | / |
| threads | Set the number of CPU threads. | 1 |
| Gene_db | The directory for the target genes DIAMOND database. | / |
| USCG_db | The directory for universal single-copy ribosomal genes database (e.g., 'Ribo_14.dmnd'). If you have already calculated the RPKM for these genes, you may instead specify the directory for the results (e.g., 'sample_name.USCG.hits.txt') to skip this step. | / |
| skip_fastp | If you have already filtered the reads, you can set T to skip running fastp. The default is to run fastp. | False |
| min_length | Set the minimum length required for filtering reads, the default is 50 bp. | 50 |
| run_seqkit | If you have already counted the total number of reads using seqkit, you can specify the directory of the seqkit results (e.g., 'sample_name.all.reads.txt') to skip running seqkit. | True |
| filter_condition | Minimum threshold of identity coverage in alignment. | Identity = 50  coverage = 80 |
| keep_samples | Whether to retain all the output results. | False |

**Supplementary Figure S3.** Format of filter condition

The uscg_rpkm() function only calculates the RPKM abundance of USCGs in the sample, and the parameter settings are shown in Supplementary Table S2; with the uscg_rpkm2GAM() function, the gene set RPKM abundance table provided by the user is converted into the gene set GAM abundance table, and the parameter settings are shown in Supplementary Table S3. The two functions are suitable for the case where there is no target gene database. The geneset.RPKM.txt and RUSCG.txt formats are shown in Supplementary Figure S4 and Figure S5, respectively.

Code example 1：

uscg_rpkm(input_reads = "your/reads/data/sample_1.fastq", result = "sample_1", threads = 40, USCG_db = "your/database/Ribo_14.dmnd", skip_fastp = F, min_length = 75, run_seqkit = T, keep_samples = F)

Code example 2：


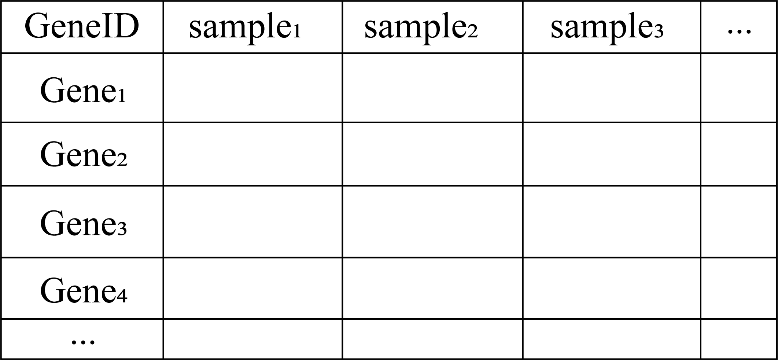
uscg_rpkm2GAM(input_geneset = "your/data/geneset.RPKM.txt", input_uscg_rpkm = "your/database/RUSCG.txt", output = "geneset.GAM.txt")

**
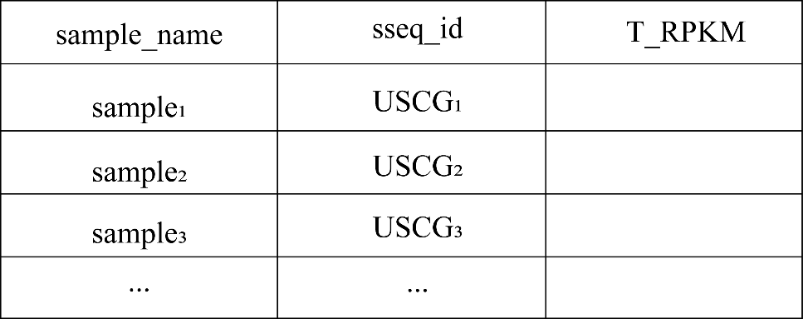
Supplementary Figure S4.** Format of geneset.RPKM.txt

**Supplementary Figure S5.** Format of RUSCG.txt

**Supplementary Table S2.** Parameters setting of uscg_rpkm

| Parameter | Description | Default |
| --- | --- | --- |
| input_reads | Set the reads to include only the forward reads if the data is paired-end (PE). | / |
| result | Set the name for the result file. | / |
| threads | Set the number of CPU threads. | 1 |
| USCG_db | The directory for universal single-copy ribosomal genes database (e.g., 'Ribo_14.dmnd'). If you have already calculated the RPKM for these genes, you may instead specify the directory for the results (e.g., 'sample_name.USCG.hits.txt') to skip this step. | / |
| skip_fastp | If you have already filtered the reads, you can set T to skip running fastp. The default is to run fastp. | False |
| min_length | Set the minimum length required for filtering reads, the default is 50 bp. | 50 |
| run_seqkit | If you have already counted the total number of reads using seqkit, you can specify the directory of the seqkit results (e.g., 'sample_name.all.reads.txt') to skip running seqkit. | True |
| keep_samples | Whether to retain all the output results. | False |

**Supplementary Table S3.** Parameter setting of uscg_rpkm2GAM

| Parameter | Description | Default |
| --- | --- | --- |
| input_geneset | File path of RPKM table of geneset. | / |
| input_uscg_rpkm | File path of RPKM table of USCG. | / |
| output | File path of result. | / |

**Supplementary Note 2**

We selected a total of 18 metagenomic samples from human intestine, ruminant rumen, freshwater, ocean, hydrothermal sediments, and glaciers, and included multiple sequencing depths of 5-60 G ( three metagenomic samples were selected for each ecological environment ), as shown in Supplementary Table S4.

| **Sample** | **Resource** | **Eniveronment** | **Sequencing platform** | **Sequencing layout** | **Sequnencing depth** |
| --- | --- | --- | --- | --- | --- |
| SRR22901851  SRR22901852  SRR22901853 | Differential responses of the gut microbiome  and resistome to antibiotic exposures in infants and adults (Li, et al., 2023). | Human gut | Illumina NovaSeq 6000 | PAIRED | 10 G |
| SRR21073084  SRR21073085  SRR21073087 | Distinct microbial hydrogen and reductant disposal pathways explain interbreed variations in ruminant methane yield (Li, et al., 2024). | Ruminant rumen | Illumina MiSeq | PAIRED | 10 G |
| SRR27489776  SRR10955139  SRR27489781 | Two decades of bacterial ecology and evolution in a freshwater lake (Rohwer, et al., 2025). | Freshwater | Illumina NovaSeq 6000 | PAIRED | 30 G |
| SRR12889303  SRR12889304  SRR12889305 | Microbial production and consumption of hydrocarbons in the global ocean (Love, et al., 2021). | Marine | Illumina HiSeq 4000 | PAIRED | 5 G |
| SRR6301445  SRR6301446  SRR6301447 | Genomic insights into potential interdependencies in microbial hydrocarbon and nutrient cycling in hydrothermal sediments (Dombrowski, et al., 2017). | Sediments | Illumina HiSeq 2500 | PAIRED | 60 G |
| SRR21686403  SRR21686404  SRR21686405 | Metagenomics reveals global-scale contrasts in nitrogen cycling and cyanobacterial light-harvesting mechanisms in glacier cryoconite (Chai, et al., 2024). | Glacier | Illumina NovaSeq 6000 | PAIRED | 20 G |

**Supplementary Table S4.** Information of samples.

The coefficient of variation of RPKM of USCG in all samples were calculated using GCompip, KOfamScan, eggNOG-mapper and HUMAnN3, respectively, as shown in Supplementary Table S5.

**Supplementary Table S5.** The coefficient of variation of 14 USCGs’ read counts (in RPKM) for each sample calculated by GCompip, KOfamScan, eggNOG-mapper and HUMAnN3.

| **Sample** | **GCompip** | **KOfamScan** | **EggNOG-mapper** | **HUMAnN3** |
| --- | --- | --- | --- | --- |
| Rumen1 | 0.140 | 0.306 | 0.326 | 0.662 |
| Rumen2 | 0.137 | 0.293 | 0.319 | 0.657 |
| Rumen3 | 0.166 | 0.298 | 0.314 | 0.625 |
| Human1 | 0.122 | 0.214 | 0.221 | 0.302 |
| Human2 | 0.118 | 0.214 | 0.216 | 0.394 |
| Human3 | 0.118 | 0.219 | 0.221 | 0.358 |
| Freshwater1 | 0.110 | 0.350 | 0.361 | 0.531 |
| Freshwater2 | 0.110 | 0.322 | 0.330 | 0.496 |
| Freshwater3 | 0.109 | 0.326 | 0.336 | 0.697 |
| Marine1 | 0.106 | 0.306 | 0.361 | 0.211 |
| Marine2 | 0.134 | 0.273 | 0.277 | 0.211 |
| Marine3 | 0.203 | 0.251 | 0.255 | 0.565 |
| Glacier1 | 0.125 | 0.304 | 0.321 | 0.546 |
| Glacier2 | 0.115 | 0.364 | 0.389 | 0.660 |
| Glacier3 | 0.105 | 0.335 | 0.337 | 0.255 |
| Sediment1 | 0.067 | 0.288 | 0.230 | 0.267 |
| Sediment2 | 0.126 | 0.253 | 0.268 | 0.493 |
| Sediment3 | 0.122 | 0.234 | 0.193 | 0.545 |

**Reference**

Chai, J.*, et al.* Metagenomics reveals the temporal dynamics of the rumen resistome and microbiome in goat kids. *Microbiome* 2024;12(1):14.

Danecek, P.*, et al.* Twelve years of SAMtools and BCFtools. *GigaScience* 2021;10(2).

Dombrowski, N.*, et al.* Genomic insights into potential interdependencies in microbial hydrocarbon and nutrient cycling in hydrothermal sediments. *Microbiome* 2017;5(1):106.

Hyatt, D.*, et al.* Prodigal: prokaryotic gene recognition and translation initiation site identification. *BMC Bioinformatics* 2010;11(1):119.

Langmead, B. and Salzberg, S.L. Fast gapped-read alignment with Bowtie 2. *Nature Methods* 2012;9(4):357-359.

Li, D.*, et al.* MEGAHIT: an ultra-fast single-node solution for large and complex metagenomics assembly via succinct de Bruijn graph. *Bioinformatics* 2015;31(10):1674-1676.

Li, H. and Durbin, R. Fast and accurate short read alignment with Burrows–Wheeler transform. *Bioinformatics* 2009;25(14):1754-1760.

Li, Q.*, et al.* Distinct microbial hydrogen and reductant disposal pathways explain interbreed variations in ruminant methane yield. *The ISME Journal* 2024;18(1).

Li, W. and Godzik, A. Cd-hit: a fast program for clustering and comparing large sets of protein or nucleotide sequences. *Bioinformatics* 2006;22(13):1658-1659.

Li, X.*, et al.* Differential responses of the gut microbiome and resistome to antibiotic exposures in infants and adults. *Nat Commun* 2023;14(1):8526.

Love, C.R.*, et al.* Microbial production and consumption of hydrocarbons in the global ocean. *Nat Microbiol* 2021;6(4):489-498.

Rohwer, R.R.*, et al.* Two decades of bacterial ecology and evolution in a freshwater lake. *Nat Microbiol* 2025;10(1):246-257.

Shen, W.*, et al.* SeqKit: A Cross-Platform and Ultrafast Toolkit for FASTA/Q File Manipulation. *PLoS One* 2016;11(10):e0163962.
